# Harnessing machine learning and multivariate analysis to explore global trends in *Cannabis sativa* research

**DOI:** 10.1186/s42238-026-00397-w

**Published:** 2026-02-21

**Authors:** Javier De La Hoz-M, Karime Montes-Escobar, Carlos Alfredo Salas-Macias

**Affiliations:** 1 Universidad del Magdalena, Santa Marta, 470004 Colombia; 2 Laboratorio Funcionamiento de Agroecosistemas y Cambio Climático – FAGROCLIM. Departamento de Ciencias Agrícolas, Facultad de Ingeniería Agrícola, Universidad Técnica de Manabí, Portoviejo, 130150 Ecuador; 3 Departamento de Formación y Desarrollo Científico en Ingeniería, Facultad de Ingeniería, Ciencia y Tecnología, Universidad Bernardo O’Hig Gins, Santiago, Chile; 4 Laboratorio Funcionamiento de Agroecosistemas y Cambio Climático – FAGROCLIM. Departamento de Ciencias Agronómicas. Facultad de Ingenierías Agroambientales, Universidad Técnica de Manabí, Portoviejo, 131302 Ecuador

**Keywords:** Cannabinoids, Bibliometric analysis, Collaborative networks, HJ-Biplot, Latent topics

## Abstract

This study employs advanced data science techniques to explore global research trends in *Cannabis sativa* from 1974 to 2024. This research integrated bibliographic datasets from PubMed, Scopus, and Web of Science. By combining latent Dirichlet allocation (LDA) and HJ-Biplot methods, we extracted actionable insights from large-scale data to address the current gap in long-term global research monitoring. The analysis identified key research topics, geographic disparities, and temporal trends, providing a comprehensive overview of the evolution of *Cannabis sativa* studies. The results highlight an increasing focus on the medical applications of *Cannabis sativa*, particularly in North America and Europe, while highlighting research gaps in emerging regions such as Africa and South America. Furthermore, the integration of multivariate methods with machine learning offers a replicable framework for managing large bibliographic datasets and enhancing data-driven decision-making in research management. Additionally, combining topic modeling with multivariate visualization provides a novel framework to understand how research themes evolve and interact. This approach serves as a strategic tool for stakeholders navigating the rapidly changing cannabis field.

## Introduction

*Cannabis sativa*, a plant with a rich history of human use, has been cultivated since the Neolithic period because of its diverse applications. It has been utilized for food, medicine, and recreational and spiritual rituals (Charitos et al. 2021; Kuddus et al. 2013; Small 2017). It is a versatile crop plant with a wide range of agricultural and industrial applications, including the production of paper, wood, and fiber and its potential use in the medicinal and pharmaceutical industries (Hussain et al. 2021).

The strict prohibition of cannabis cultivation for recreational, medical, and industrial purposes severely restricted scientific research for decades. Due to these stringent regulations, the plant's potential for drug discovery remained largely unexplored until its medical use was legalized, starting in California and subsequently in numerous countries worldwide (Hussain et al. 2021).

Given the growing number of publications on *C. sativa*, multiple bibliometric reviews have been conducted in recent years, reflecting the global interest in the therapeutic properties and industrial applications of this plant. However, much of the previous research has been limited in scope, focusing on specific thematic areas, narrow geographical contexts, or traditional methodological approaches. This study employed a novel methodology that comprehensively covered global research on *C. sativa*. By integrating advanced machine learning techniques with multivariate visualization, we provided an exhaustive and detailed analysis of research trends and international collaborations.

Over the years, several studies have attempted to analyze the evolution of cannabis research, each with its approaches and limitations. In 2016, Osca-Lluch et al. (2016) published a study examining 1,008 documents on drug abuse or substance use in the Web of Science database. Although interest in this field increased, its focus was limited to topics related to substance abuse, overlooking other critical areas of cannabis research, that same year, Yeung et al. (2019) analyzed the 100 most-cited articles on the endocannabinoid system, cannabis, and cannabinoids, with a focus on research published between 1986 and 2016. This study provided insight into the most influential work up to that time, and its focus on publications prior to 2016 excluded recent advancements in this rapidly evolving field.

Later, in 2017, Matielo et al. (2018) analyzed six decades of cannabis publications, using specific keywords to study the biochemistry, genetics, and traceability of the plant, although they offered an in-depth view of certain areas, the use of limited keywords restricted the scope of their analysis, leaving broader research areas such as industrial applications or the ecological impacts of cannabis, in 2019, Liu et al. (2021) addressed the global evolution of research on cannabis and cannabidiol, covering publications from 1940–2019. Despite their broad temporal coverage, this study focused on the chemistry, pharmacology, and molecular biology of cannabidiol, excluding other aspects of *C. sativa*.

Ng and Chang (2022) published a bibliometric analysis of nearly 30,000 documents related to cannabis and cannabinoids. Although they provided a broad overview, their methodology was restricted to title searches, which reduced the depth of their analysis. Similarly, Anokwuru et al. (2022) focused exclusively on cannabigerol; however, by limiting their bibliometric review to this single compound, they omitted other significant aspects of C. sativa research. Finally, Zurián et al. (2021) investigated the 100 most-cited articles on addictions to cannabis and other psychoactive substances, providing a narrow view focused on addictions without covering other potential uses of the plant.

More recently, in 2023, Sixto-Costoya et al. (2023) presented a study analyzing the evolution of marijuana research from a biopsychosocial perspective, examining three thematic branches: anthropology, chemistry, and psychiatry; however, their focus on specific terms such as “marijuana use” and “marijuana abuse” resulted in limited data retrieval; similarly, Díaz-Bárcena et al. (2023) conducted a comparative analysis of *Papaver somniferum* and *C. sativa*.

Mano-Sousa et al. (2024) conducted a comprehensive bibliometric analysis of more than 10,000 publications on *C. sativa*, identifying the most influential authors and countries in the field. Although the study covered both medical and recreational uses of cannabis, its primary focus was on pharmacology, with less exploration of areas such as molecular biology, genetics, or industrial applications. Finally, in 2024, Laaboudi et al. (2024) published an analysis of cannabis research in Morocco between 2012 and 2022. This work highlighted the significant growth of research on the benefits of cannabis in Morocco, with a focus on medicine, pharmacology, and neuroscience, although it was more focused on international collaborations and less focused on emerging areas such as the ecological impact of cannabis cultivation.

Throughout this evolution, it has become clear that most previous studies have focused on specific or limited research areas on *C. sativa*, whether in geographic, thematic, or temporal terms. Previous studies focused primarily on medical aspects, substance abuse, or recreational use. However, they failed to integrate emerging areas such as industrial applications and environmental impact. Furthermore, these studies lacked advanced analytical tools capable of providing a comprehensive view of the global research landscape.

This study differentiates itself from previous studies by offering a broader and more comprehensive view of global research on *C. sativa*, overcoming the limitations observed in earlier works. Rather than focusing on specific thematic areas or limited geographical regions, this analysis provides a global view that includes both medical and industrial and ecological uses of the plant.

This research integrates state-of-the-art data science techniques, including latent Dirichlet allocation (LDA) (Blei et al. 2003) and HJ biplot (Galindo 1986), to uncover global research trends in *Cannabis sativa*. These methods enable the analysis of large-scale structured and unstructured data, providing a comprehensive understanding of thematic clusters, geographic disparities, and temporal trends. By combining machine learning with multivariate analysis, this study provided a replicable framework for managing bibliographic data. This approach supports evidence-based decision-making and helps identify emerging areas of interest in research management.

Furthermore, this paper contributes to the information management literature by demonstrating how advanced analytical methods can enhance the organization, analysis, and dissemination of complex datasets. The findings have practical implications for policymakers, researchers, and institutions aiming to allocate resources strategically and foster global collaboration in *Cannabis sativa* research.

## Materials and methods

### Search strategy and data collection

**Fig. 1:**
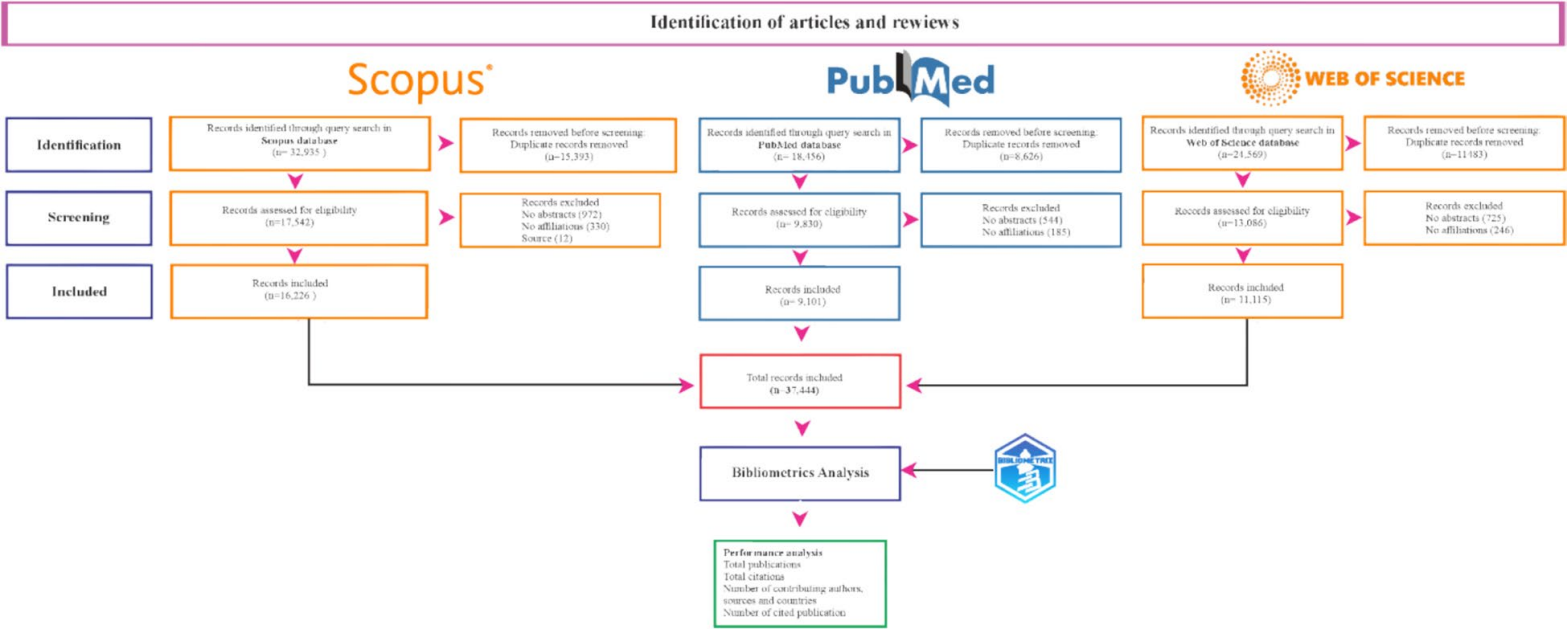
Flowchart of the data selection and filtering process

To ensure transparency and reproducibility in the data collection process, a structured selection and filtering workflow was implemented. The initial search was conducted on September 8, 2024, using the Scopus database. Since the research aimed for a comprehensive global overview, the search strategy included the terms 'cannabis sativa', 'marijuana', and 'marihuana', utilizing Boolean operators to encompass all relevant variants (Table 1). The systematic filtering of records, from initial identification to final inclusion, is detailed in the flowchart presented in Fig. 1.

**Table 1:** Bibliographic databases and keywords

Bibliographic database	Search data	Search string	Results
Scopus	September 8, 2024	TITLE-ABS-KEY ("cannabis sativa" OR marihuana OR marijuana) AND PUBYEAR > 1973 AND PUBYEAR < 2025 AND (LIMIT-TO (DOCTYPE, "ar") OR LIMIT-TO (DOCTYPE, "re")) AND (LIMIT-TO (SRCTYPE, "j"))	*N* = 32,935
Web of Science	September 8, 2024	TS = ("cannabis sativa" OR marihuana OR marijuana)	*N* = 24,569
PubMed	September 8, 2024	("cannabis sativa" OR marihuana OR marijuana[Title/Abstract]) AND "journal article"[Publication Type] AND 1974/01/01:2024/09/08[Date—Publication]	*N* = 18,456

The data were obtained from three primary databases frequently utilized by researchers: PubMed, Scopus, and Web of Science (WoS). Both Scopus and WoS are widely used databases in various scientific fields for literature search and citation analysis; they offer comprehensive coverage of scholarly literature, including journals, conference proceedings, patents, and other types of publications; researchers often use these databases to find relevant literature, track citations, and assess the impact of their research (Pranckutė 2021).

Scopus is known for its broad coverage of scientific disciplines, including the natural sciences, social sciences, health sciences, and humanities. Moreover, WoS covers a wide range of academic disciplines, including the sciences, social sciences, arts, and humanities (Pranckutė 2021; Chadegani et al. 2013). Both databases have strengths and weaknesses, and researchers often use them in combination to ensure comprehensive coverage of the literature relevant to their research (Pranckutė 2021; Chadegani et al. 2013). However, PubMed is a web portal for the MEDLINE medical database, developed by the National Center for Biotechnology Information (NCBI), this institute, specializing in biotechnology data processing, is part of the U.S., Department of Health and Human Services, and PubMed hosts approximately 20 million citations of MEDLINE biomedical literature, as well as articles from biological sciences journals and online books (Tober 2011).

Integrating the three datasets can present challenges, especially due to variations in article information depending on the source, whether it is from PubMed, Scopus, or Web of Science. To unify the data collected from each of these sources, the Bibliometrix (Aria and Cuccurullo 2017) package in R was used, which includes a specific function for merging files from different databases.

During the process of unifying the databases, 35,501 duplicate documents, 2,241 documents without abstracts, seven without sources, seven without titles, and 760 documents without affiliation information were removed. As a result, the final dataset was consolidated into 37,444 documents (Fig. 1).

The collected metadata included title, abstract, publication year, source, and author affiliation.

### Bibliometric analysis

Bibliometrics, a quantitative method used for analyzing academic literature by examining bibliographies, offers a means of describing, evaluating, and monitoring published work. Different bibliometric methods are applicable to specific research inquiries, and scientific mapping can be achieved by addressing common questions via bibliometrics (Aria and Cuccurullo 2017). In this study, we adopted an objective and reliable approach, considering four levels of analysis: countries, sources, documents, and authors.

The retrieved bibliographic information has enabled us to conduct a quantitative analysis to obtain a comprehensive overview of scientific production within the scope of our study. This encompasses document distribution, literature production dynamics, and prolific sources such as researchers, institutions, countries, and sources.

We utilized the bibliometrix R-Tool (Aria and Cuccurullo 2017), an R package (R Core Team. R 2023) that provides specialized tools for quantitative bibliometric and scientometric research to facilitate this analysis.

### Topic model

Topic modeling is an unsupervised machine learning method employed to automatically identify the topic or topics present in a single document or a collection of documents. This technique usually reveals latent or hidden topics that are not directly mentioned in the texts. These underlying topics are represented by clusters of words that are frequently used together to describe a concept, and these words often appear in similar linguistic contexts (DiMaggio et al. 2013).

We utilized Latent Dirichlet Allocation (LDA), a robust unsupervised machine learning algorithm, to identify latent thematic structures within the corpus. This probabilistic technique, rooted in Bayesian modeling, serves as a sophisticated extension of Probabilistic Latent Semantic Analysis (Blei et al. 2003; Hornik and Grün 2011). The core principle of the LDA model is that each document is treated as a random mixture of latent topics, where each topic is defined by a specific distribution of words from the global vocabulary (Blei et al. 2003). Unlike supervised approaches, this unsupervised method enables the discovery of themes without prior labeling, assuming a fixed number of topics distributed across the collection. In this model, each document addresses multiple themes, and every term is assigned a probability of belonging to a particular topic. This document-topic probability distribution provides the essential data structure for subsequent multivariate analysis and longitudinal trend mapping.

The objective of LDA is to infer or estimate latent variables by calculating their conditional distribution relative to the documents. Equation (1) illustrates the statistical assumptions underlying the generative process of LDA.

1 $$\begin{array}{ll}p\left(\beta_K,\theta_D,z_D,w_D\right)\\=\prod\limits_{k=1}^Kp\left(\beta_K\vert\eta\right)\prod\limits_{m=1}^Mp\left(\theta_m\vert\alpha\right)\prod\limits_{n=1}^Np\\\left(z_{m,n}\vert\theta_m\right)P\left(w_{m,n}\vert z_{m,n},\beta_{m,k}\right)\end{array}$$

In this context, K, M, and N represent the number of topics, articles, and words in a given document, respectively. The parameters α and η (Dirichlet hyperparameters) define the prior distributions over θ and β. Here, θ_m_ denotes the distribution of topics for article m (real vector of length *K*); *z*_m,*n*_ is the topic assigned to the *n*-*t*ℎ word in the m-*t*ℎ article; and *w*_m,*n*_ represents the *n*-th word of the m-*t*ℎ document. Additionally, β_*k*_ describes the word distribution for topic *k*. To uncover the hidden structure, we must condition the only observable variable—the words within the documents—using statistical inference methods. The conditional probability, also referred to as the posterior probability, is defined by Eq. 2.

2 $$p\left(\beta_K,\theta_M,z_M\vert w_M\right)=\frac{p\left(\beta_K,\theta_M,z_M,w_M\right)}{p\left(w_M\right)}$$

Although the exact computation of the posterior probability is impossible because the denominator term (Blei et al. 2003), it can be approximated via statistical posterior inference methods. There are two primary inference techniques: variational-based algorithms (Wang and Blei 2011; Blei and Jordan 2006) and sampling-based algorithms (Porteous et al. 2008). An example of a sampling-based algorithm is the Gibbs sampler (Griffiths and Steyvers 2004). Both variational- and sampling-based algorithms yield similarly accurate results (Asuncion et al. 2012).

#### Identifying research topics

The process of identifying topics via LDA was divided into three stages: (i) preprocessing, (ii) creation of the LDA model, and (iii) topic labeling. The data processing for this part of the study was conducted via LDAShiny (Fernández-Gómez and Mendes 2021), an open-source package for the R programming language. This package includes a tool that offers a web-based graphical user interface, allowing for the review of scientific literature through the Bayesian approach of latent Dirichlet allocation (LDA) and machine learning algorithms.

#### Preprocessing texts

The downloaded articles underwent a series of essential preprocessing operations. This phase, referred to as 'text refining' by Tang et al. (2014), involved transforming the documents into a standardized format suitable for subsequent analysis.

To enhance the coherence of the topics, each abstract was tokenized using bigrams, which are combinations of consecutive unigrams. Although this may seem straightforward, the process requires converting the text into lowercase and removing punctuation marks, dashes, brackets, numbers, spaces, and other unnecessary characters. Additionally, a standard list of words known as "stopwords", which primarily serve to make a sentence grammatically correct (e.g., articles and prepositions), was identified and removed.

#### Creation model latent Dirichlet allocation

Topic models are hidden variables that use correlations between words and underlying semantic themes within a document collection (Blei and Lafferty 2007). This approach requires specifying the expected number of topics *k* (i.e., latent variables) in advance. Selecting the appropriate number of topics for a given set of articles is a complex task. Different strategies have been employed to address this challenge, aiming to strike a balance between having enough topics to cover the entire document collection and keeping the number manageable to ensure that the results remain interpretable. This study conducted simulations by varying k from 2–40 in increments of one, using an inference algorithm with 500 iterations, specifically Gibbs sampling (Geman and Geman 1984). We evaluated the LDA model quality using a topic coherence measure (Röder et al. 2015). This metric assesses the model based on human interpretability, which is considered more appropriate than computational measures like perplexity (Chang et al. 2009).

#### Labeling topics

The topics generated by the LDA model require semantic labeling, as algorithmic analyses often face significant challenges in capturing the nuanced meanings of human language. Consequently, manual labeling is widely regarded as the standard practice in topic modeling (Han Lau et al. 2011).

To ensure an accurate and meaningful interpretation, the topics were manually labeled using two primary sources of information: the lists of the most frequent (or most likely) words associated with each topic and a selection of article titles accompanied by summaries of the three most representative articles.

The selection of these three articles per topic was guided by their high probability of association with the corresponding topic, as determined by the LDA model. These articles served as the most relevant and thematically aligned examples, providing clear and focused insights into the core characteristics of each topic. Limiting the selection to three articles allowed for a concise yet comprehensive representation of the topics, maintaining a balance between analytical depth and interpretability without overwhelming the analysis or the reader.

To visualize the relationships between the identified topics and their relative distances, we employed the LDAvis package (Sievert et al. 2014) in R. This tool generates an intertopic distance map via multidimensional scaling (MDS), providing an intuitive two-dimensional representation of how topics are interconnected based on shared terms. In the map, each topic is represented by a circle, whose size indicates the topic's prevalence in the corpus. The distance between circles represents the semantic similarity between topics.

#### Quantitative indices used to analyze the trends of topics

Given the enormous volume of articles and the extensive number of words, understanding topics and their trends intuitively can be challenging. To solve the problem, several quantitative indices proposed by Xiong et al*.* (2019), were utilized. These indices, derived by aggregating document-topic and topic-word distributions, help clarify the results and findings. Their description is as follows: the distribution of topics over time is obtained by

3 $${\theta }_{k}^{y}=\frac{{\sum }_{m\epsilon y}{\theta }_{mk}}{{n}^{y}}$$

In this context, $m\epsilon y$ represents the articles published in a specific year, ${\theta }_{mk}$ denotes the proportion of the k-th topic in each article, and ${n}^{y}$ is the total number of articles published in that year (Xiong et al. 2019).

The distribution of topics across journals is defined as the proportion of the k-th opic within a given journal j:${\theta }_{k}^{j}$

4 $${\theta }_{k}^{j}=\frac{{\sum }_{m\epsilon j}{\theta }_{mk}}{{n}^{j}}$$

In this context, $m\epsilon j$ refers to the articles within a specific journal, ${\theta }_{mk}$ represents the proportion of the k-th topic in each article, and ${n}^{j}$ represents the total number of articles published in journal j.

The distribution of topics across countries is defined as the proportion of the k-th topic within country c:

5 $${\theta }_{k}^{c}=\frac{{\sum }_{m\epsilon c}{\theta }_{mk}}{{n}^{c}}$$

where mϵc represents the articles from a specific country, θ_mk_ indicates the proportion of the k-th topic in each article, and n^c^ refers to the total number of articles published in country c.

To facilitate the characterization of topics in terms of their trends, we employed simple regression slopes for each topic, with the year as the independent variable and the proportion of the topics in that year as the response variable (Griffiths et al. 2004). The topics identified through this regression analysis were classified as having either positive or negative trends, depending on whether the slope was statistically significant at the 0.01 level.

### HJ biplot

Biplots are visual tools used to represent multivariate data, enabling the display of three or more variables, much like a scatter plot shows the combined distribution of two variables. Originally introduced by Gabriel (1971), the Biplot method has evolved into various specialized techniques, such as the JK-Biplot and the GH-Biplot. While the JK-Biplot focuses on analyzing similarities between rows (individuals), the GH-Biplot is designed to explore correlations between columns (variables). To simultaneously optimize the representation of both rows and columns within the same low-dimensional space, the HJ-Biplot (Villardón 1986) was developed. The literature (Escobar et al. 2021; Pilacuan-Bonete et al. 2022; Montes-Escobar et al. 2023) demonstrates the application of this method to enhance the analysis of data generated by the latent Dirichlet allocation (LDA) model.

The interpretation of the HJ-Biplot integrates concepts from factor analysis, multidimensional scaling, and correspondence analysis (Villardón 1986). In this framework, the lengths of the column vectors represent the standard deviation of the variables, while the distances between row markers reflect their proximity or similarity. Furthermore, the angles between vectors provide a direct measure of correlation: acute angles indicate a strong positive correlation, obtuse angles signify a negative correlation, and right angles suggest that variables are uncorrelated.

## Results

### Bibliometric analysis

Table 2 summarizes the results from the review of articles on *C. sativa* published between 1974 and 2024. In total, 37,444 documents were identified, originating from 6,694 different sources. The annual growth rate of 5.16% reflects sustained growth in scientific output. The average age of the documents is 11.2 years, while each document receives an average of 32.7 citations, highlighting continuous and significant interest in the topic.

**Table 2:** Main information about the bibliometric analysis of research on *Cannabis sativa*

Description	Results
Main Information about Data	
Timespan	1974:2024
Sources (Journals)	6,694
Documents	37,444
Annual Growth Rate %	5.16
Document Average Age	11.2
Average Citations Per Doc	32.7
Document Contents	
Keywords Plus (Id)	50,158
Author's Keywords (De)	40,086
Authors	
Authors	77,176
Authors Of Single-Authored Docs	2307
Authors Collaboration	
Single-Authored Docs	2960
Co-Authors Per Doc	4.76
Document Types	
Article	33,138
Review	4,306

For the authors, 77,176 researchers participated, of whom 2,307 published individually. However, most studies resulted from collaboration, with an average of 4.76 coauthors per document. Regarding document types, original research articles (33,138) were the most common, followed by reviews (4,306). This shows that original research articles are more prevalent than literature reviews in this field of study.

**Fig. 2:**
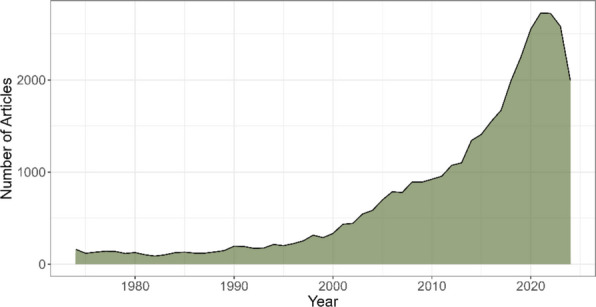
Annual scientific production of *Cannabis sativa* from 37,444 articles published between 1974 and 2024

The trend analysis of publications on *C. sativa* between 1974 and 2024 revealed marked growth, with several distinguishable phases. During the first period, from 1974 to 1990, a relatively stable trend was observed, with slight fluctuations in the number of annual publications, ranging between 88 and 195 articles (Fig. 2). During this time, publications remained moderate, possibly reflecting less interest or stricter regulatory restrictions regarding the study of this plant.

However, starting in 1990, the number of articles began to rise, with steady growth from 1996 onward. This increase may be associated with growing research on the potential medicinal uses of cannabis and greater social and scientific acceptance. This shift is reflected in the consistent increase in publications, reaching over 300 articles per year by 2000 and exceeding 500 articles per year by 2004. From 2005 onward, the acceleration in the number of publications became even more notable, with exponential growth culminating in 2021, with 2727 articles.

The highest point in publications was reached in 2021, coinciding with the boom in clinical research and the approval of more favorable legislation in several countries for the medical and recreational use of cannabis. This scientific peak is characterized by a diversification of research topics. For instance, recent studies have significantly contributed to understanding marijuana dependence (Rich et al. 2024; Ryerson et al. 2024) and its psychological implications (Coelho et al. 2025; Litt et al. 2023; Wellman et al. 2022). Furthermore, the medical field has seen a surge in pharmacological and behavioral research (Berey et al. 2023; Smith et al. 2024; Martin-Willett et al. 2023), alongside specialized studies in cannabinoid properties (Awal et al. 2022; Nickles and Lio 2020). Finally, the forensic field has also adapted to this trend, focusing on new detection methods and genetic identification (Cisana et al. 2024; Di Nunzio et al. 2021, 2024). These works illustrate the multidimensional nature of the 2021 production spike, moving beyond basic botany into complex clinical and legal applications.

**Fig. 3:**
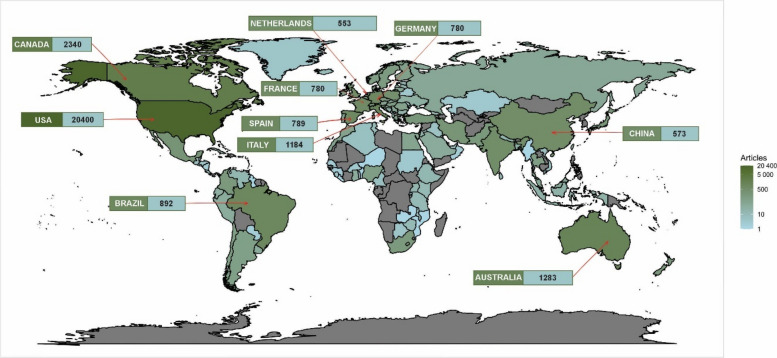
Distribution of geographical origins in the analysis of 37444 published articles on *Cannabis sativa* from 1974 to 2024

The geographic distribution of publications in Fig. 3 shows that a precise concentration is observed in a small group of countries. The United States stands out as the leading producer, with 20,400 publications, representing a substantial portion of the scientific literature in this field; Canada follows it (2,340 publications), Australia (1,283), Italy (1,184), Brazil (892), and Spain (789), which make up the six countries with the highest production.

Europe plays an important role in *C. sativa* research, with notable contributions from Germany (780), France (660), the Netherlands (553), the United Kingdom (537), and Poland (398). Asia also has a significant presence, with China (573), India (506), and Israel (424) leading the research in the region. In Latin America, Brazil stands out as the largest producer, followed by Mexico (279), Colombia (177), and Chile (119), reflecting growing interest in this area of study.

Despite the predominance of developed countries in terms of scientific output, some emerging countries also show considerable activity, such as Turkey (228), South Africa (187), and the Czech Republic (179). However, in many regions of Africa, the Middle East, and Asia, the volume of publications is significantly lower, with countries such as Egypt (69), Nigeria (115), and Morocco (78) showing more limited contributions.

At the lower end of the scale, numerous countries, such as Zimbabwe (10), Hong Kong (9), and Palestine (8), have fewer than ten publications, highlighting disparities in research capacity across different regions. This uneven distribution reflects the scientific infrastructure and resource gap between developed and developing countries.

Table 3 shows that research on *C. sativa* is primarily concentrated in journals specializing in addictions, toxicology, and drug policy, with notable growth in more specialized publications in recent years. The high number of citations and h-indices in several of these journals underscores their impact on the scientific understanding of cannabis in various areas of health and society. *Drug and alcohol dependence* tops the list with 1,330 publications, an h-index of 108, and 55,636 citations have accumulated since 1975, establishing itself as the leading source in this field. This list is followed by *Addictive Behaviors* with 980 publications and 32,973 citations, reflecting its influence on cannabis research, with an h-index of 86 since 1980.

**Table 3:** Top 30 scientific journals for research on *Cannabis sativa*. H = h-index, TC = total citations, NP = number of publications, and PY_start = year of publication start. The table is organized in descending order by NP

Source	NP	TC	h_index	PY_start
Drug and Alcohol Dependence	1330	55,636	108	1975
Addictive Behaviors	980	32,973	86	1980
Substance Use & Misuse	543	7930	42	1999
Addiction	494	33,548	99	1993
Psychology of Addictive Behaviors	322	13,236	64	1993
American Journal of Drug and Alcohol Abuse	309	8790	51	1974
Psychopharmacology	296	15,581	70	1976
Journal of Studies on Alcohol and Drugs	289	6881	44	2007
Journal of Adolescent Health	286	17,231	74	1991
Plos One	274	7085	46	2006
International Journal of Drug Policy	273	6540	43	1999
Industrial Crops and Products	261	10,256	57	1992
Substance Use and Misuse	259	5511	36	1974
Journal of Drug Issues	251	5154	39	1974
Journal of Psychoactive Drugs	227	5405	37	1987
Cannabis and Cannabinoid Research	218	3724	31	2016
American Journal On Addictions	189	5000	39	1997
Journal of Substance Abuse Treatment	180	6019	40	1984
International Journal of Environmental Research and Public Health	168	1905	19	2009
Cureus Journal of Medical Science	168	619	11	2017
Drug and Alcohol Review	165	4476	33	1990
Experimental and Clinical Psychopharmacology	159	4219	34	1995
Journal of Drug Education	159	2249	27	1974
Molecules	150	3315	31	2014
Substance Abuse	149	2828	30	1995
Pediatrics	146	13,027	64	1980
Journal of Analytical Toxicology	142	5574	42	1977
Forensic Science International	142	4563	39	1978
Journal of Substance Use	141	717	14	1997
Neurotoxicology and Teratology	137	9306	54	1987

In third place is *Substance Use & Misuse,* with 543 publications, followed by *Addiction* and *Psychology of Addictive Behaviors,* with 494 and 322 publications, respectively. Notably, *Addiction* has an h-index of 99, indicating a relatively high impact compared with other journals.

Among more recent journals, *Cannabis and Cannabinoid Research*, founded in 2016, has quickly positioned itself with 218 publications and an h-index of 31, emerging as a key source in cannabis and cannabinoid-specific research; similarly, the *Cureus Journal of Medical Science*, despite having a more modest h-index (11), has published 168 articles since its founding in 2017.

Another relevant journal is *Plos One*, which has published 274 articles since 2006 and has an h-index of 46 and 7,085 citations, highlighting its crucial role in disseminating open-access research across various disciplines.

Lastly, toxicology and forensic analysis journals like the *Journal of Analytical Toxicology* and *Forensic Science International* are crucial for finding and judging compounds that come from cannabis. Their h-indexes of 42 and 39 are especially noteworthy.

### Latent Dirichlet allocation

The LDA model with the optimal coherence score comprises 30 topics (k = 30). Table 4 provides a thematic classification of 37,444 articles published on *C. sativa* between 1974 and 2024, identifying 30 main topics along with the number of associated publications (NP) for each, with the topics ranging from cardiovascular and respiratory health to social aspects and cannabis production.

**Table 4:** Topics discovered from 37,444 articles published on *Cannabis sativa* between 1974 and 2024. NP = number of publications

T	Label	top_terms	NP
t_1	Cardiovascular and Respiratory Health	diseas, acut, death, bodi, cardiovascular, injuri, lung, mortal, pressur, respiratori, lead, blood, histori, chronic, syndrom	700
t_2	Anxiety and Depression Disorders	symptom, depress, anxieti, class, stress, psycholog, affect, relationship, moder, examin, emot, sever, neg, level, associ	960
t_3	Adolescents and Substance Use	adolesc, substanc, school, youth, parent, alcohol, prevent, famili, behavior, peer, factor, risk, grade, examin, initi	3499
t_4	Risk and Protective Factors	risk, associ, ag, factor, model, regress, odd, adjust, examin, analys, variabl, logist, data, initi, logist_regress	1526
t_5	Genetics and Cannabis Research	gene, analysi, genet, identifi, type, profil, sequenc, specif, environment, express, reveal, cluster, strain, differenti, dna	739
t_6	Brain Reward Mechanisms	brain, respons, control, activ, alter, region, function, reward, imag, chronic, volum, effect, rat, process, structur	964
t_7	Illicit Drugs and Abuse	drug, cocain, illicit, abus, illicit_drug, alcohol, drug_abus, heroin, addict, alcohol_drug, amphetamin, methamphetamin, illeg, ecstasi, drug_user	1787
t_8	Social Perceptions of Cannabis	posit, neg, perceiv, alcohol, social, consequ, motiv, drive, influenc, percept, expect, attitud, measur, person, driver	884
t_9	Patterns of Cannabis Use	user, compar, frequent, daili, differ, frequenc, pattern, regular, heavi, report, current, nonus, user_user, occasion, consumpt	288
t_10	Systematic Reviews on Cannabis	studi, review, evid, includ, effect, literatur, data, search, systemat, identifi, limit, conduct, base, databas, evalu	914
t_11	Cannabinoid Receptor Studies	cannabinoid, receptor, cb, activ, effect, endocannabinoid, potenti, synthet, mechan, role, human, compound, therapeut, cannabinoid_receptor, cb_receptor	2461
t_12	Clinical Attention to Cannabis Users	patient, screen, hospit, care, clinic, posit, primari, center, report, visit, conclus, emerg, includ, score, compar	926
t_13	Mental Health Disorders	disord, substanc, depend, abus, mental, alcohol, health, mental_health, treatment, substanc_abus, psychiatr, individu, substanc_disord, sud, addict	1359
t_14	Policies and Legalization Trends	legal, polici, recreat, health, public, product, law, harm, market, impact, public_health, access, regul, consum, unit	1879
t_15	Trends in Cannabis Consumption	increas, time, rate, decreas, cud, period, level, trend, chang, observ, declin, compar, differ, data, remain	147
t_16	Cannabis Cultivation and Production	plant, hemp, product, yield, speci, industri, crop, cultiv, growth, flower, field, cultivar, soil, grow, leav	1946
t_17	Clinical Cannabis Interventions	particip, dai, intervent, month, outcom, assess, baselin, control, follow, reduc, measur, week, complet, report, suicid	1095
t_18	Hemp Extracts and Benefits	treatment, effect, clinic, sleep, trial, efficaci, advers, improv, therapi, treat, qualiti, evid, potenti, withdraw, control	858
t_19	THC and CBD Studies	hemp, extract, acid, oil, content, seed, properti, composit, materi, product, fiber, food, process, chemic, compound	2045
t_20	Cognition and Psychosis	thc, cbd, delta, effect, dose, tetrahydrocannabinol, cannabidiol, administr, concentr, delta_tetrahydrocannabinol, δ, delta_thc, tetrahydrocannabinol_thc, cannabinoid, subject	1906
t_21	Ethnicity, Gender, and Cannabis	cognit, control, function, perform, impair, subject, memori, psychosi, schizophrenia, attent, effect, task, term, measur, psychot	964
t_22	Pain Management and Opioids	american, white, differ, gender, ethnic, black, race, hispan, african, african_american, statu, examin, commun, educ, urban	477
t_23	Smoking, Alcohol, and Cannabis	medic, pain, patient, opioid, medicin, chronic, cancer, prescript, condit, manag, provid, report, care, physician, purpos	1411
t_24	Cannabis Detection Methods	smoke, alcohol, tobacco, cigarett, student, drink, consumpt, colleg, smoker, nicotin, current, dai, univers, vape, bing	1812
t_25	Prenatal Cannabis Exposure	sampl, method, test, detect, analysi, determin, urin, valid, posit, sensit, mass, standard, rang, collect, perform	1049
t_26	Cannabis Surveys and Data	exposur, women, pregnanc, children, prenat, expos, matern, ag, mother, birth, outcom, pregnant, infant, childhood, child	893
t_27	Social Development and Cannabis	report, survei, adult, preval, data, ag, nation, health, popul, sampl, cross, respond, method, conclus, section	851
t_28	Cannabis and Sexual Behavior	develop, social, discuss, approach, base, strategi, provid, commun, understand, peopl, explor, support, experi, practic, paper	1181
t_29	Cellular Effects and Inflammation	behavior, risk, sexual, sex, femal, male, health, hiv, report, risk_behavior, physic, violenc, partner, behavior, male_femal	1315
t_30	Youth and Cannabis Risks	cell, activ, level, beta, induc, inhibit, reduc, anti, alpha, inflammatori, express, treat, respons, vitro, diseas	608

Adolescent substance use stands out among the most addressed topics, with 3,499 publications (t_3). This topic includes key terms related to youth, substance use, and risk factors associated with consumption. It is followed by studies on cannabinoid receptors, with 2,461 publications (t_11), which examine biological mechanisms and the role of cannabinoids in the human body. Studies on THC and CBD (t_19) are also prominent, with 2,045 publications covering research on the most well-known chemical compounds of cannabis.

Another significant topic is legalization and public policy concerning cannabis (t_14), with 1,879 publications addressing changes in legislation and their impact on public health. Additionally, research on cannabis cultivation and production (t_16) includes 1,946 publications, highlighting aspects such as plant growth and crop yields.

There is also considerable focus on pain management and opioid use (t_23), with 1,411 publications reflecting interest in the therapeutic potential of cannabis for chronic pain management. Studies related to cannabis detection methods (t_24), with 1,812 publications, are also prominent and focus on techniques for detecting cannabis use and other substances.

At the lower end of the list are topics such as cellular and inflammatory effects (t_29), with 608 publications, and cardiovascular and respiratory health (t_1), with 700 publications, examining the body's biological responses to cannabis and its impact on specific diseases. Overall, this thematic classification provides a comprehensive overview of the breadth of research on *C. sativa*, highlighting its social and legal implications as well as its effects on health and well-being.

**Fig. 4:**
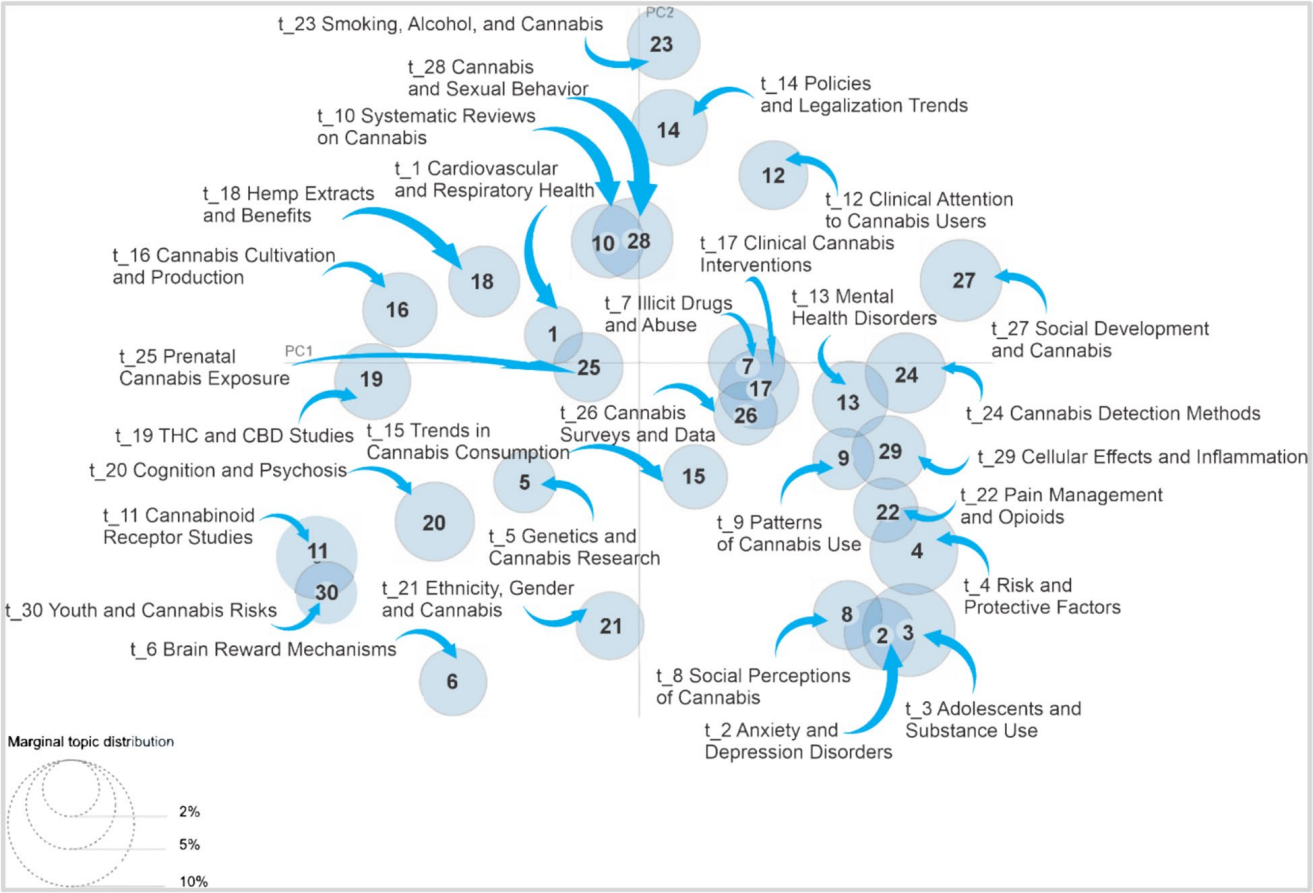
Intertopic distance map. *Cannabis sativa* (1974 to 2024)

Figure 4 visualizes the relationship between the identified topics in the corpus. In this type of representation, each circle corresponds to a topic, and its location on the plane reflects thematic similarity: the closer two circles are, the more closely related their subject matter is; conversely, those that are more distanced address divergent themes, and the size of the circles is directly related to the prevalence of each topic in the analyzed set of documents.

The map reveals several clusters of topics with clear thematic connections; for example, Mental Health Disorders and Patterns of Cannabis Use are very close, suggesting a strong relationship in the literature between cannabis use and mental health, Similarly, studies on drug abuse and clinical cannabis interventions are grouped together. This shows that studies on drug abuse are closely linked to medical interventions in the cannabis context.

On the other hand, some topics appear more isolated. Brain Reward Mechanisms, one of the largest on the map, is positioned far from others, indicating that it is a widely researched subject but has fewer direct connections to other major topics. This suggests that studies on brain reward mechanisms form an autonomous body of research within the global thematic set.

The map also helps to identify more specialized or emerging research areas, topics related to Social Perceptions of Cannabis and Risk and Protective Factors are moderately connected but remain distant from other thematic clusters, suggesting that these research lines may be developing independently, without strong ties to the rest of the analyzed topics.

Additionally, Cannabinoid Receptor Studies, which appears close to subjects such as THC and CBD, might reflect growing interest in biochemical and pharmacological research on the effects of cannabis, particularly regarding its most well-known components.

The Intertopic Distance Map not only provides a visual representation of how cannabis research topics are clustered but also offers a tool to identify emerging trends and less connected research areas.

**Fig. 5:**
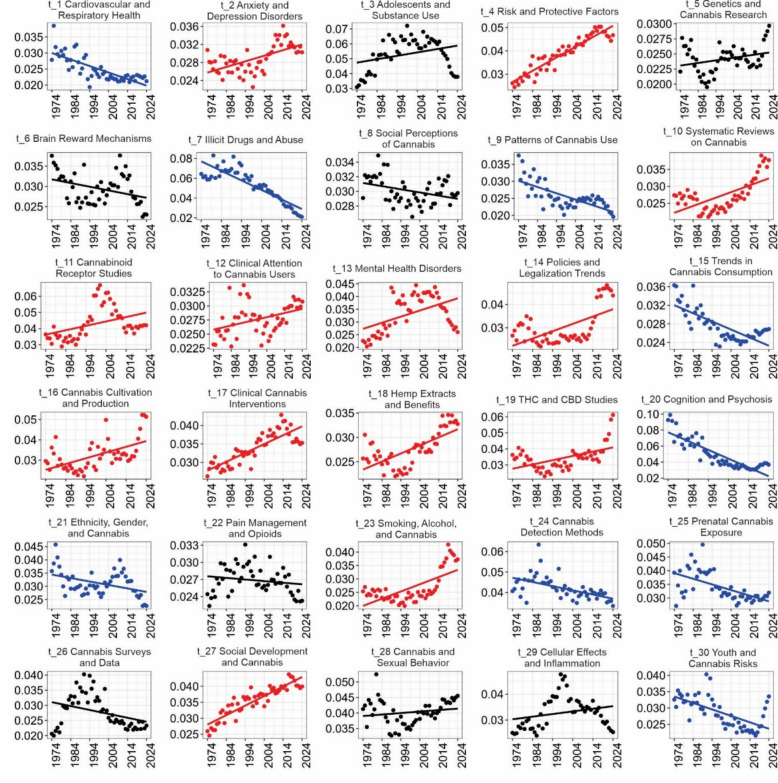
Trends of topics from 37444 *Cannabis sativa* articles published between 1974 and 2024. The red line indicates topics with an increasing trend, the blue line indicates a decreasing trend, and the black line represents fluctuations without a marked trend

Figure 5 illustrates the temporal trends of 30 topics derived from articles published between 1974 and 2024. Trajectories are color-coded to reflect their evolution: red lines indicate growth, blue lines signify a decline, and black lines represent fluctuating patterns without a clear direction. Notably, 17 topics show an increasing trend, suggesting a rise in scientific and public interest. This growth is likely driven by ongoing debates regarding cannabis policy, as well as its medicinal and clinical applications. Six topics display a decreasing trend, indicating a decline in publication frequency and research focus, and seven topics show a fluctuating trend, with no clear upward or downward trajectory.

### Hj-Biplot

**Fig. 6:**
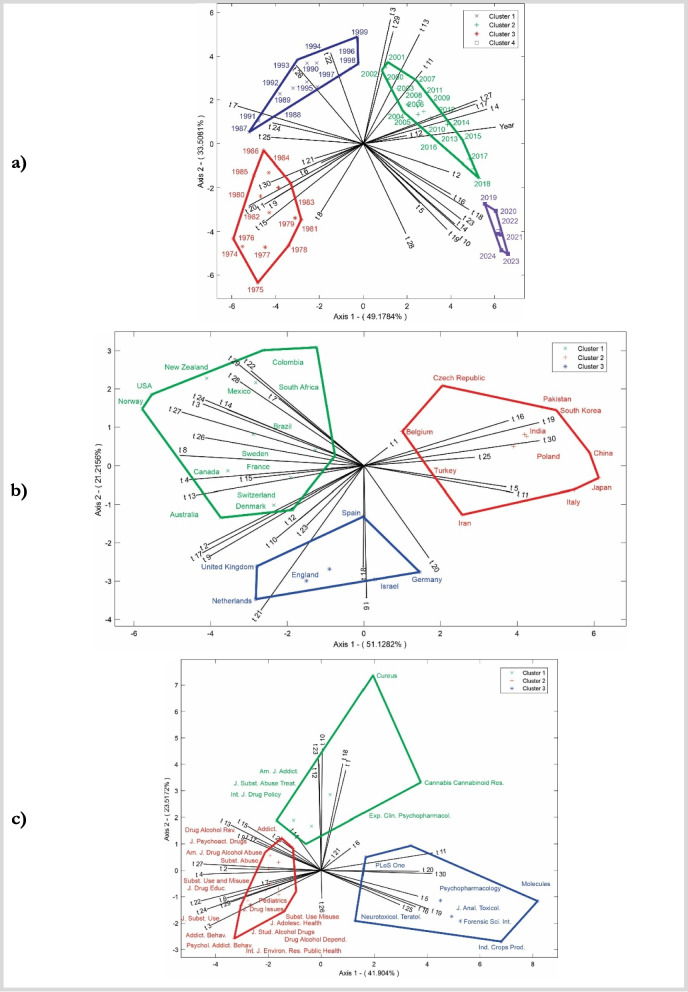
Associations among topics per year (**a**), topics per country (**c**) and topics per journal (**b)** via the HJ-Biplot method

The analysis examined temporal, periodical, and geographical dimensions via multivariate HJ-Biplot, resulting in theta matrix outputs, with probability coefficients for each matrix ranging from 0–1. As shown in Fig. 6, the year-based chart captures 82.69% of the total variability, the country-based chart explains 72.34%, and the journal-based chart accounts for 65.41%. Together, these graphs provide a significant and coherent representation of the relationships between topics, years, countries, and journals.

Figure 6a shows the red group, representing early cannabis research from 1974 to 1986. During this period, studies focused primarily on the risks to youth and the effects on cardiovascular and respiratory health. Researchers also analyzed consumption patterns and emerging trends in cannabis use. These years reflected growing concerns about the risks associated with cannabis consumption, particularly among younger populations, and an urgent need to understand the long-term health consequences of using this substance. In the blue group, covering the years 1987–1999, cannabis research shifted to its impact on health and prenatal development. Studies have addressed topics such as pain management through the use of opioids and cannabis, as well as methods to detect the presence of cannabis. Special attention has been given to the effects of cannabis on pregnant women and the risks it poses to fetal development. This period marked advancements in understanding how cannabis affects both clinical outcomes and exposure detection across different contexts. For the green group, which spans from 2000–2018, research has increasingly focused on mental health disorders such as anxiety and depression in relation to cannabis use. Studies have also explored clinical interventions using cannabis, cannabinoid receptor research, and its impact on adolescents. Additionally, risk and protective factors for users have been examined. This period marked a significant rise in clinical and scientific attention to cannabis use in various medical conditions, emphasizing its impact on mental health and youth development. Finally, the purple group, covering the years 2019–2024, reflects research centered on specific cannabis compounds such as THC and CBD and their therapeutic applications. There has also been a strong focus on legalization policies and the benefits of hemp extracts. These studies signal a shift toward broader acceptance of cannabis in medical and commercial fields, with increasing interest in its components and the evolving legal frameworks surrounding its use worldwide.

Figure 6b shows the green group, comprising countries such as the United States, Canada, Brazil, Australia, Mexico, Norway, New Zealand, Colombia, South Africa, Sweden, France, Switzerland, and Denmark. These countries focus on research that explores the social and mental health aspects of cannabis; key topics include cannabis consumption patterns, social perceptions, and the impacts of legalization policies. Additionally, this group emphasizes population data collection through surveys and studies on risk and protective factors related to cannabis use, with a notable focus on public health implications and regulatory issues. The red group, which includes countries such as China, India, Iran, Turkey, Japan, Italy, Poland, South Korea, Pakistan, the Czech Republic, and Belgium, centers on scientific and clinical studies related to cannabis genetics; the effects of THC and CBD; and impacts on cardiovascular and respiratory health. These countries also prioritize research on the risks of cannabis use among youth, prenatal exposure, and cannabinoid receptors. This cluster strongly focuses on the physiological effects of cannabis, combining advances in genetic research with clinical studies. Finally, the blue group, composed of Spain, England, the Netherlands, Germany, and Israel, distinguishes itself through a focus on systematic reviews of cannabis, as well as studies on cannabis production and cultivation. This cluster also investigates sociocultural differences related to cannabis use and explores the relationships between ethnicity, gender, and consumption patterns; additionally, they examine the combined use of tobacco, alcohol, and cannabis. These countries tend to adopt a multidisciplinary approach, integrating social and cultural aspects with cannabis production and regulation.

Figure 6c shows the green group, which focuses on research related to cardiovascular and respiratory health, clinical care, and interventions for cannabis users. Journals such as the American Journal of Addiction and Journal of Substance Abuse Treatment emphasize on clinical treatments and policies on substance abuse. Whereas Cannabis Cannabinoid Research and Experimental and Clinical Psychopharmacology are journal specialized in the clinical and pharmacological mechanisms of cannabinoids. Clinical interventions, brain reward mechanisms, and factors associated with alcohol, tobacco, and cannabis use are some of the most important topics. This shows that there is a strong link between pharmacology and addiction treatment. Key topics include clinical interventions, brain reward mechanisms, and factors associated with alcohol, tobacco, and cannabis use, highlighting a strong link between pharmacology and addiction treatment. The red group focuses on mental health disorders and psychological well-being, particularly among adolescents and substance users. This cluster includes journals such as Drug and Alcohol Review, Addiction, and Pediatrics, addressing issues such as illicit drug abuse, risk and protective factors, and social development linked to cannabis use. The topics covered span a wide range of concerns, including anxiety, depression, pain management, and social perceptions of substance use, indicating a focus on the prevention and treatment of drug misuse in social and mental health contexts. Finally, the blue group covers studies on the biological and toxicological effects of cannabis, as well as research on the plant's genetic and chemical components. Journals such as PLoS One, Psychopharmacology, and Molecules focus on cannabinoid receptor studies; THC and CBD effects; and the impact of cannabis on cognitive and psychological development, especially in young people. This cluster also includes research on prenatal cannabis exposure, cellular effects, and inflammation, with a strong focus on the biological mechanisms and long-term effects of cannabis at the cellular and molecular levels.

## Discussion

This research highlights the transformative potential of machine learning and multivariate analysis in information management. By applying LDA, the study revealed 30 latent thematic structures, whereas the HJ-Biplot method provided a multivariate perspective on the relationships between topics, regions, and temporal patterns. These techniques enable the effective organization and analysis of bibliographic datasets, supporting data-driven decision-making in resource allocation and research planning. Compared with traditional approaches, this methodology significantly expanded the scope and depth of the analysis. Unlike the studies by Ng and Chang (2022) and Sixto-Costoya et al. (2023), which restricted their focus to specific areas like the endocannabinoid system, our approach addresses these limitations through a meticulously designed systematic search across PubMed, Scopus, and Web of Science, ensuring the retrieval of peer-reviewed articles without geographic or thematic restrictions between 1974 and 2024.

The analysis of trends reveals significant patterns that reflect the evolution of scientific and societal priorities. A consistent increase in publication volume has been observed, peaking in 2021, driven by the growing acceptance of therapeutic uses and legislative reforms (Ng and Chang 2022; Sixto-Costoya et al. 2023). This scientific peak is characterized by a diversification of research topics; for instance, recent studies have significantly contributed to understanding marijuana dependence (Rich et al. 2024; Ryerson et al. 2024) and its psychological implications (Coelho et al. 2025; Litt et al. 2023; Wellman et al. 2022). Furthermore, the medical field has seen a surge in pharmacological and behavioral research (Berey et al. 2023; Smith et al. 2024; Martin-Willett et al. 2023), alongside specialized studies in cannabinoid properties (Awal et al. 2022; Nickles and Lio 2020). Finally, the forensic field has also adapted to this trend, focusing on new detection methods and genetic identification (Cisana et al. 2024; Di Nunzio et al. 2021, 2024). These works illustrate a transition from research focused primarily on consumption risks to studies addressing clinical and legal applications, such as chronic pain management and the benefits of cannabinoids (t_11, t_19, t_23).

Despite this growth, the present analysis reveals significant gaps. Emerging areas, such as the ecological impact of cultivation (t_16) and cellular and inflammatory effects (t_29), remain underdeveloped. This thematic imbalance contrasts with extensive research in fields such as adolescent use (t_3) or clinical interventions (t_17), highlighting the need for more diversified research integrating biomedical, ecological, and industrial perspectives. Future studies should explore how C. sativa compounds interact at the cellular level and offer innovative solutions to mitigate the environmental effects of its production through interdisciplinary methodologies.

Furthermore, the findings expose a stark geographic imbalance: countries such as the United States, Canada, Australia, and Italy consistently lead research, whereas regions such as Africa, Latin America, and parts of Asia contribute relatively less. These disparities are largely influenced by differences in infrastructure, financial resources, and regulatory policies. However, underrepresentation from certain regions does not necessarily indicate a lack of research potential, but rather a lack of opportunities and institutional support. For example, many developing nations possess unique ecological conditions suitable for industrial and medicinal studies but lack the means to scale their efforts effectively.

As noted by Laaboudi et al. (2024) and Mano-Sousa et al. (2024), international collaborations demonstrate the potential to address these gaps. Strengthening partnerships between high-output countries and those with limited contributions—through technology transfer programs, capacity-building workshops, and increased access to shared databases—is essential for ensuring equitable advancements.

While this analysis provides a comprehensive perspective, its reliance on databases such as Scopus, WoS, and PubMed may have excluded gray literature or local publications. Additionally, the exclusion of nonrelevant documents (e.g., books, gray literature, and reports) contributed to maintaining a high signal-to-noise ratio but may have limited the inclusion of alternative relevant perspectives. Finally, the need for manual interpretation in topic labeling may introduce subjective biases. These aspects could be improved in future studies by employing more advanced automated techniques and incorporating more diverse data.

To build upon these findings, future research should expand the data scope by incorporating multilingual datasets and alternative outputs, such as gray literature and patent data, which would mitigate potential database biases. Methodologically, the integration of network analysis could further enrich the understanding of collaboration networks and knowledge flows within the Cannabis sativa research community. Moreover, the application of advanced techniques—including temporal topic modeling and deep learning—could enhance the granularity of insights, allowing for a more precise examination of how legal and regulatory changes continue to reshape the dynamic evolution of this field.

## Data Availability

The dataset supporting the conclusions of this article is available in the ZENODO repository at the following link: [10.5281/zenodo.14715411].
